# Exploring deep reinforcement learning acceleration by superscaling data augmentation via branched fractal symmetries

**DOI:** 10.3389/frobt.2026.1791812

**Published:** 2026-07-01

**Authors:** Ryan Vander Stelt, Cleiver Ruiz-Martinez, Caeden Rosen, Blake Hull, Juan Rojas

**Affiliations:** 1 Computer Science (CS), Lipscomb University, Nashville, TN, United States; 2 Electrical Engineering and Computer Engineering (EECE), Lipscomb University, Nashville, TN, United States; 3 Mechanical Engineering, Lipscomb University, Nashville, TN, United States

**Keywords:** sample-efficient, reinforcement learning, superscaling, fractal symmetry, physical robot learning, on-robot learning, data augmentation

## Abstract

Learning deep reinforcement learning (DRL) policies directly in physical robots remains bottlenecked by slow wall-clock training times. We present preliminary research on *Branched Euclidean Group Fractal Symmetries*, a trajectory-level augmentation framework that super-scales group transformations to accelerate policy learning for manipulation. We model a Markov decision process (MDP) as a tree of state–action pairs; at each depth, affine transformations generate geometric structures, within which combinations of Euclidean group symmetries produce a large set of unique symmetric equivalences. A self-similar branching rule can also be iterated across depths to yield fractal symmetry expansions that populate the replay buffer with diverse yet consistent experiences with Cartesian or hand-centered image representations. We show the efficacy of our approach through simulated and real Franka robot manipulation contact tasks. Our study learns robust policies directly in physical robots in as fast as 14 min and in 20 min of wall-clock time on average. The code is accessible at https://github.com/lippyRobotics/fractalserl.

## 1 Introduction

Learning deep reinforcement learning (DRL) policies directly from physical robots is a desirable goal as robots can learn world physics and avoid sim2real pitfalls. Previous research has leveraged symmetry principles for data augmentation, transforming robot interactions with the physical world via invariant transformations to accelerate learning. Researchers have also introduced invariant layers directly into neural network architectures. SOTA work can learn DRL-based robot manipulation policies in 30–120 min, depending on the task, typically with approximately 20 demonstrations to bootstrap the algorithm. These learning speeds, however, are not yet fast enough for robots to be practically deployed and enjoy wider adoption in unstructured environments. One limitation includes the limited number of extracted symmetries from the original interaction.

**FIGURE 1:**
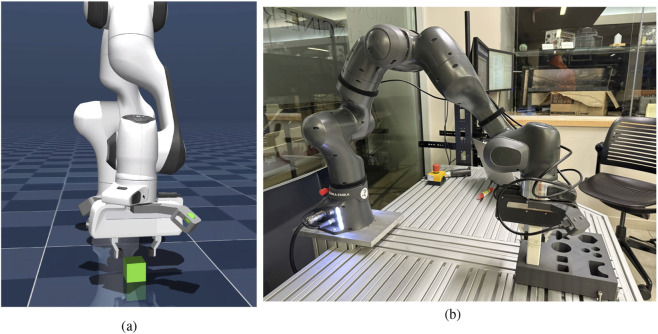
Simulated and real Franka Research Robot 3 examples of manipulation contact tasks. **(a)** Simulated Franka performs a reach task. **(b)** Franka robot performs a peg-insert task with random resets.

In this study, we present Fractal SERL, *preliminary research* on fractal symmetry. We demonstrate how branched symmetries accelerate DRL policy learning in physical robots by super-scaling robot data generation. This data augmentation significantly speeds up the learning of DRL policies directly on physical robots while improving policy performance and consistency. Figure 1 depicts a contact-rich peg insertion task used in this study. Along with Fractal SERL, a highly efficient data store and replay buffer implementation was conceived to support efficient parallelized computations and image handling without excessive memory usage. Finally, we introduce the normalized area under the curve (nAUC) as a trajectory-wide performance metric to capture the combined contributions of sample efficiency and performance throughout the policy’s lifetime, which have yet to be used to assess the comparative performance of DRL policies.

**FIGURE 2:**
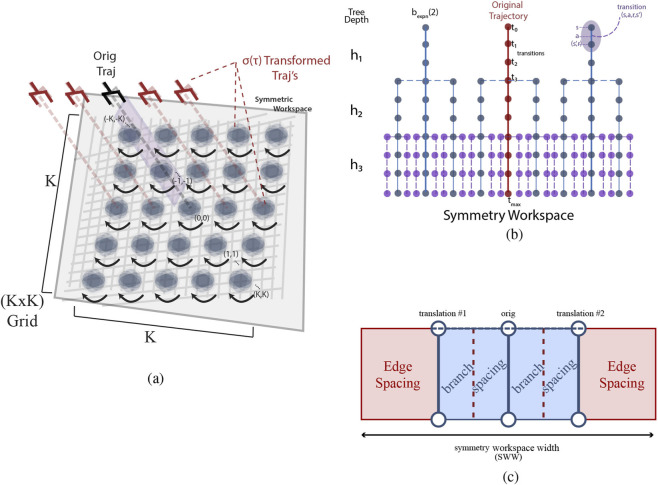
Branching strategy framework. **(a)** Fractal grid enclosed by the blue blobs. The original trajectory (states and actions) is subjected to multiple transformations at each depth. With each transformation, a K×K grid of transformed states (vertices) and actions (edges) in the tree is generated. **(b)** Cross-section of the fractal branching system with a branching factor of 2 (plus the original trajectory). Child nodes remain within the “symmetry workspace width.” **(c)** Depiction of spacing across a cross-section of the grid for three branches (inclusive).

We conceive a Markov decision process (MDP) as a tree in which an episodic trajectory, composed of states and actions, is encoded by vertices and edges, respectively. An affine transformation can be used on the trajectory to transform state–action (vertex–edge) pairs at a particular depth. These affine transformations can be applied to generate geometric structures such as a grid, as shown in Figure 2a. Within this grid, Euclidean group symmetries (translations, reflections, and rotations) can be applied in various combinations to produce a superset of unique symmetric equivalencies at a given time-step. Furthermore, at each new depth, a self-similar branching rule can be applied to produce *new branches* within the geometric structure (see Figure 2b). By iterating, a fractal symmetry is generated.

## 2 Related literature

We review contributions that speed up DRL policies via symmetry-based concepts in fully observable settings. Invariant Transform Experience Replay (ITER) (Lin et al., 2020) transformed trajectory information $\left(s,a,r,s^{\prime }\right)$ via reflectional symmetry and trained a deep deterministic policy gradient. ITER produced up to 15 new reflectional symmetries for reach, slide, and pick-and-place tasks and achieved a 3×–13× improvement in sample efficiency relative to Hindsight Experience Replay (HER) baseline (Andrychowicz et al., 2017). Wang et al. (2021) embedded a cyclic group of dimension 12 $C_{12}$ to encode rotational symmetry directly in a fully connected network (FCN). The FCN was convolved with an image, producing an action heatmap to instantiate picks. The system produced 3× sample efficiency improvements against RAD (Laskin et al., 2020) and DrQ (Kostrikov et al., 2020). According to Wang et al. (2022b), SO(2)-equivariant RL defined an equivariant Q-function and an invariant optimal policy, leading to the production of Equi-DQN and Equi-SAC, respectively. These used depth images and displacements and were tested with a number of algorithms, achieving significant performance gains and approximately a 10× improvement in sample efficiency. According to Wang et al. (2022a), SO(2)-equivariant RL learned online via equivariant learning, and Equi-SAC used a dihedral group $D_{4}$ in the NN and an augmentation of four random SO(2) samples to train across block picking, clutter grasping, block pushing, and block-in-bowl. In this study, a UR5 robot was trained between 45 min and 2 h 40 min of wall-clock time for meaningful performance.

The presented methods use few symmetries in their data generation process. We explore the use of branched symmetries to *super-scale* the generation of possible symmetries and their effect on improving wall-clock training times, sample efficiency, performance, and robustness.

## 3 Preliminaries

### 3.1 Fractal branching systems

Fractals or branched symmetries pervade nature, offering simple rules for building complex, efficient structures across multiple scales. Consider the racemose plant, especially the corymb inflorescence, which branches radially alongside the stem to produce a flat-topped or convex crown. We draw inspiration from such systems in our study.

Fractal and branching systems (a trivial fractal) can be modeled via iterated function systems (IFSs) (Hutchinson, 1981). Consider a tree G with vertices V , edges E , and depth h such that G=(V,E) . The tree has spatial geometric properties and branching dynamics we can model.

Consider a vertex $v_{k}$ , which describes a pose of interest $x\in R^{n}$ at a depth $h_{j}$ . Vertices or branches (these will be used interchangeably) $v_{k}$ can be produced via an affine transformation $T_{k}\left(x\right)=Ax+t_{k}$ , which belongs to an n -dimensional Euclidean group E(n)∈(A,t) . The transformation A∈O(n) is a member of the orthogonal group and captures rotations and reflections, and $t\in R^{n}$ is a translation acting on x . Such vertices can be arranged to form a desirable structure such as a linear branched system, a planar K×K grid, or more complex geometries (polygons, stars, etc.). Figure 2a shows an example of a grid structure.

A branching rule b determines how each vertex branches when activated. We consider four branching strategies: (i) a constant branching strategy (the trivial fractal) $b_{\mathrm{const}}\left(0\right)$ , where the starting grid does not change throughout the episode; (ii) an expanding branching strategy, with a branching factor f : $b_{\mathrm{expn}}\left(f\right)$ ; (iii) a contracting branching strategy $b_{\mathrm{cntr}}^{*}\left(f\right)$ ; and (iv) a dissociated branching strategy that lets branches grow or shrink in an unrelated manner—*e.g.,* an hourglass shape.

All vertices $\left(v_{1},\ldots ,v_{k}\right)$ at a particular depth $h_{j}$ are encompassed by a set $V_{h}$ . The IFS, which models fractal symmetry, is summarized by the Hutchinson operator acting on the starting set $V_{0}$ , the fixed point, to produce the same compact set^1^ under the generative process (Hutchinson, 1981): $H\left(V\right)={⋃}_{k}^{H}T_{k}\left(V\right)$ .

### 3.2 Markov decision processes

We model an MDP via the tuple ⟨S,A,P,R,p,γ⟩ , where S is a continuous state space, A is a continuous action space, P:S×A×S→[0,1] is the unknown transition function that describes the environmental dynamics $p\left(s^{\prime }|s,a\right)$ , $R\left(s,a,s^{\prime }\right)$ is the reward when an agent reaches state $s^{\prime }\in S$ after performing action a∈A in state s∈S and can be dense or sparse, $p\left(s_{0}\right)$ is the probability distribution over initial states, and γ∈[0,1] is a discount factor. The continuous robot learning problem corresponds to the RL problem of obtaining a parameterized stochastic policy $\pi_{\theta }:S\to P\left(A\right)$ that maps states to a distribution of actions, such that the expected maximum-entropy objective is maximized for any given goal (Haarnoja et al., 2018).

### 3.3 SERL

SERL (Luo et al., 2024a) is a software suite for sample-efficient robotic reinforcement learning. It is composed of sample-efficient off-policy DRL methods, a variety of reward generation methods (sparse and dense, Cartesian and image-based via binary classifiers and adversarial techniques), environment reset methodologies for reset-free physical training, a safe RL high-quality impedance controller, and a number of challenging tasks that converge on state-of-the-art results that average between 30 and 105 min. We select to build our fractal branching on this framework.

## 4 Branched Markov decision processes

Branched MDPs can super-scale robot learning by leveraging invariant transformations in RL rollouts. Consider an episodic RL setup with a trajectory τ of finite duration $t_{max}$ as sequences of state, action, reward tuples: $\langle s_{0}$ , $a_{1}$ , $r_{1}$ , $s_{1}$ , $a_{2}$ , $r_{2}$ , $s_{2},\ldots ,s_{t_{\max}}\rangle$ . For a branched MDP, each vertex represents a state s∈S and each edge represents an action a∈A . We define the set of all executed (contact-rich) trajectories as $\overset{¯}{\Gamma }=\cup_{h=1}^{H}S\times {\left(A\times R\times S\right)}^{h}$ . A global affine symmetrical transformation σ applied to each contact-rich feasible trajectory is an invariant one-to-one mapping $\sigma :\overset{¯}{\Gamma }\to \overset{¯}{\Gamma }$ that generates a newly formed physical rollout: $\sigma \left(\overset{¯}{\Gamma }\right)=\left\{\tau^{\prime }\in \overset{¯}{\Gamma }|\exists \tau \in \overset{¯}{\Gamma },\sigma \left(\tau \right)=\tau^{\prime }\right\}$ (Lin et al., 2020). The affine transformation can be understood as a decomposable symmetry that applies to the trajectory’s states and actions: $\sigma_{S}:S\to S$ , and $\sigma_{A}:A\to A$ and are reward-preserving. The rewards (and returns) in $\tau^{\prime }$ appear exactly as in τ . The trajectory tuple becomes: $\tau^{\prime }=\langle s_{0}^{\prime },a_{1}^{\prime },r_{1}^{\prime },s_{1}^{\prime },a_{2}^{\prime },r_{2}^{\prime },s_{2}^{\prime },\ldots ,s_{t_{max}}^{\prime }\rangle$ , where the symmetry mapping is applied to each MDP element at every time step in the episode: $s_{0}^{\prime }=\sigma_{S}\left(s_{0}\right)$ , $a_{i}^{\prime }=\sigma_{A}\left(a_{i}\right)$ , $s_{i}^{\prime }=\sigma_{S}\left(s_{i}\right)$ , and $r_{i}^{\prime }=R\left(\sigma_{S}\left(s_{i-1}\right),\sigma_{A}\left(a_{i}\right),\sigma_{S}\left(s_{i}\right)\right)$ .

In Fractal SERL, the symmetry operator can be set to a reward-invariant and dynamics-invariant branched symmetry operation: $\sigma \left(\cdot \right):T_{k}\left(\cdot \right)=A\left(\cdot \right)+t_{k}$ . The transformation is applied to both states (vertices) $s_{i}^{\prime }=\sigma_{S}\left(s_{i}\right)=T_{k}\left(s_{i}\right)=A\left(s_{i}\right)+t_{k}$ and actions (edges) $a_{i}^{\prime }=\sigma_{A}\left(a_{i}\right)=T_{k}\left(a_{i}\right)=A\left(a_{i}\right)+t_{k}$ . The transformations are conducted to produce a desirable geometric branched structure (line, grid, or more complex shapes introduced in Section. 3.1), as shown in Figure 2a. The result is a finite set of transformed vertices and edges (including the original pair) at each time-step. As such, when activated, branching rules control the number of newly generated transformed transitions. Tree depth h may be equivalent to the MDP time-steps, but not necessarily (see Section 4.1.3 for more on this).

### 4.1 Branching properties

We present our branched-group symmetry methodology.

#### 4.1.1 Branching geometric structure and affine global transformations

We select affine transformations to produce a K×K grid geometric structure conformed by vertices that include the original trajectory, where K∈Z . A grid visualization is shown in Figure 2a. Note that the structure is generated immediately when the MDP starts at depth $h_{0}$ in the tree (see Section. 4.1.3 for more on depth calculations). Figure 2b depicts a cross-section of the branching scheme.

Within the E(n) affine transformation group, a combinatorial set of possible symmetries exists within the grid structure and includes translations $t_{k}$ , rotations R , and reflections FB : (i) pure translations: $\sigma_{t}\left(\overset{¯}{\Gamma }\right):I\left(\overset{¯}{\Gamma }\right)+t_{k}$ , (ii) rotations followed by translations: $\sigma_{R}\left(\overset{¯}{\Gamma }\right):R\left(\overset{¯}{\Gamma }\right)+t_{k}$ , (iii) reflections followed by translations: $\sigma_{F}\left(\overset{¯}{\Gamma }\right):F\left(\overset{¯}{\Gamma }\right)+t_{k}$ , and finally, (iv) a rotation–reflection combination followed by translations: $\sigma \left(\overset{¯}{\Gamma }\right):T\left(\overset{¯}{\Gamma }\right)+t_{k}$ . Ultimately, the symmetry operation $\sigma \left(\overset{¯}{\Gamma }\right)$ is the union of all combinations: $\sigma_{h}\left(\overset{¯}{\Gamma }\right)=\sigma_{h_{t}}\left(\overset{¯}{\Gamma }\right)⋃\sigma_{h_{R}}\left(\overset{¯}{\Gamma }\right)⋃\sigma_{h_{F}}\left(\overset{¯}{\Gamma }\right)$ .

Recall that a trajectory symmetry is decomposed into state and action transformations: $\sigma \left(\overset{¯}{\Gamma }\right):\sigma_{S}\left(\cdot \right)⋃\sigma_{A}\left(\cdot \right)$ . Care must be taken in the way that symmetries are applied, in particular to states. In our formulation, transformations are global rather than local. State representations may include proprioception (pose, twist, wrench, and finger) information, along with more complex representations (images, point clouds, etc.). For our preliminary study, we limit the problem to hand-centered images I and proprioception information. Hand-centered images are notably easier to work with than images elsewhere since their relation to end-effector proprioception remains invariant. For pure global translations $\sigma_{t}\left(I\right)=I$ , images remain invariant. For rotations and reflections, images could be transformed by the equivalent transformation: $\sigma_{R}\left(I\right)=R\left(I\right)$ and $\sigma_{F}\left(I\right)=F\left(I\right)$ (Lin et al., 2020).

Note that workspace constraints may be violated. When a transformation places any entity in the environment (e.g., the end-effector or relevant objects) outside the workspace nominal limits, such trajectories are not included in the replay buffer to ensure that the reflection is invariant.

The motivation behind the combinatorial set is to aid in super-scaling the robot interaction. Consider a 27×27 grid. A total of 729 vertices (including the original one) are spawned for each unique symmetrical transformation at a given time-step. The entire generated dataset is inserted into the replay buffer B before sampling and optimization: $\sigma \left(\overset{¯}{\Gamma }\right)\;\to \;B$ . When images are involved, our implementation creates a separate image buffer, preventing image duplication during transformations. The transition saves an index to its image, ensuring that the correct image is fetched during sampling. Without this optimization, the replay buffer capacity would be limited when Fractal SERL uses a high number of branches.

A key step in maximizing efficient learning in our Fractal SERL is to parallelize all transformations in the K×K grid. We briefly present computations for the grid translations $t_{\left(k,k\right)}\in K\times K$ along the robot’s base xy -plane. We define a square “symmetry workspace” (SymWS) within which all transformations must lie. The SymWS is constrained to exist within the larger robot workspace. The SymWS is always centered at the robot’s end-effector position. The edge corner location of the SymWS is computed by subtracting the robot’s end-effector (x,y) position from the SymWS half-width: $SymWS_{\left(0,0\right)}=s_{\left(x,y,\cdot \right)}-\frac{SymWS_{\mathrm{width}}}{2}$ .

Given this knowledge, transformations are measured from the edge of the SymWS, not the center location of the end-effector. At each end of the grid, there will always be some degree of free space, denoted as “Edge Spacing” in Figure 2c in red. This edge spacing is a function of the number of branches (inclusive of the original) at that depth (three branches in our example in Figure 2c). The branch positioning is controlled by $t_{k_{x},k_{y}}$ , where each k is an index into the grid on the xy -plane; the branch index $b_{i,j}$ , which starts at 1; and the total branch count for that depth $b_{c_{h}}$ . Iterating from one grid corner to the other, Equation 1 can be obtained:

$$t_{k_{x},k_{y}}=SymWS_{\left(0,0\right)}+\frac{\left(2*b_{i,j}-1\right)*SymWS}{2*b_{c_{h}}}.$$ (1)

Independent of this grid structure is the branching strategy that determines how vertices change with depth.

#### 4.1.2 Branching strategy

As presented in Section 3.1, we have three branching systems, each with some branching factor f : $b_{\mathrm{cnst}}\left(0\right)$ , $b_{\mathrm{expn}}\left(f\right)$ , and $b_{\mathrm{cntr}}^{*}\left(f\right)$ . The last two produce fractal systems if their branching factor remains unchanged at different depths—in effect, a generative self-similar rule necessary for fractals. Both the branching expansion and contraction can start with any K×K grid, but the former grows exponentially with depth, while the latter cuts branches. It is desirable to have the flexibility to adjust the tree’s growth or contraction in response to the given environment. Additionally, the system allows for dissociative branches, in which branching factors change with depth. Dissociative branches provide flexibility to produce varying depth/temporal-branching patterns, which could be useful in some scenarios. When entering a new tree depth $h_{j+1}$ , the number of branches will change. The location of all new vertices is controlled by the translation operation presented in Equation 1 for the grid.

#### 4.1.3 Depth methods

This section clarifies the difference between the tree depth $h_{j}$ and the time-steps $t_{\max}$ and explains how depth is set. Depth controls when new branches are produced in fractal expansions $b_{\mathrm{expn}}$ or $b_{\mathrm{cntr}}$ contractions. Care must be taken when setting depth values as the branch number can increase or decrease rapidly during an RL rollout. This has a downstream effect on learning as the number of transition transformations can significantly change the proportion of entries in the replay buffer for that transition. Maximum depth can be empirically hard-coded; if so, depth is computed as follows: $d\;\left(t+1\right)=d\;\left(t\right)+1\left(\left[t\;\mathrm{mod}\;\left[\frac{t_{\max}}{max\text{\_}depth}\right]=0\right.\right)$ . Other methodologies are possible, but they were not used during experimentation.

#### 4.1.4 Measuring performance

In DRL, when measuring acceleration during policy learning, researchers typically use pointwise metrics, for example, the number of samples to achieve a particular return or the maximum performance in the task. We present a simple yet informative metric, the nAUC, which captures both efficiency and performance. In RL return or success rate plots whose results approach the upper-left corner, their nAUC values will be closer to 1. The larger the area of one metric relative to another, the better the overall efficiency and performance.

#### 4.1.5 Pseudocode

The pseudocode for Fractal SERL is presented in Algorithm 1.

Algorithm 1 **Fractal SERL pseudocode.**

 Require: robot environment E ; optional demos $D_{0}$ ; discount γ ; UTD ratio K

 Ensure: trained policy $\pi_{\theta }$

 Initialize:
 Policy $\pi_{\theta }$ , critic $Q_{\phi }$ , target critic $Q_{\overset{¯}{\phi }}\leftarrow Q_{\phi }$

 Replay buffer $B\leftarrow D_{0}$

 Set reward model $R_{\psi }$

 while training: do
  Observe state $s_{t}∼E$

  Sample action $a_{t}∼\pi_{\theta }\left(\cdot |s_{t}\right)$

   Execute $a_{t}$ via a low-level impedance controller
   Observe next state $s_{t+1}$

   Specify reward: $r_{t}\leftarrow r\left(s_{t},a_{t}\right)$

   Compute symmetry: $\sigma \left(\overset{¯}{\Gamma }\right)$

   Store $\left(\overset{¯}{\Gamma },\sigma \left(\overset{¯}{\Gamma }\right)\right)$ in B

   for k = 1, …, K do {high update-to-data ratio}
    Sample minibatch M :
    half from $D_{0}$ , half from B

    Critic update: $y\leftarrow r+\gamma E_{a^{\prime }∼\pi_{\theta }}\left[Q_{\overset{¯}{\phi }}\left(s^{\prime },a^{\prime }\right)\right]$ $\phi \leftarrow \phi -\eta \nabla_{\phi }{\left(Q_{\phi }\left(s,a\right)-y\right)}^{2}$

    Actor update: $\theta \leftarrow \theta +\eta \nabla_{\theta }E_{a∼\pi_{\theta }}\left[Q_{\phi }\left(s,a\right)+\alpha H\left(\pi_{\theta }\left(\cdot |s\right)\right)\right]$

    Target update: $\overset{¯}{\phi }\leftarrow \tau \phi +\left(1-\tau \right)\overset{¯}{\phi }$

    end for
    if episode terminates then
     Reset environment via scripted or manual reset
     return $\pi_{\theta }$

## 5 Experiments and results

We present *preliminary results* for Fractal SERL in simulation and with a real robot. Extensive experiments have been conducted on a limited number of tasks, including reach in simulation and peg insertion in the real robot.

Three metrics are used to report results: efficiency (sample steps and wall-clock time), performance (returns or success rate), and our nAUC, which accounts for both efficiency and performance throughout the policy’s life.

### 5.1 Simulated FR3 Reach

**FIGURE 3:**
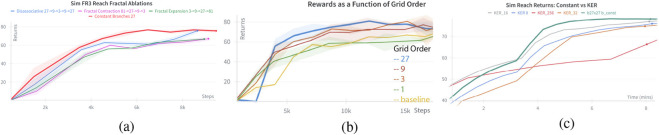
Fractal SERL results for simulated FR3 reach across five seeds. Mean shown in bold. The envelope shows one standard deviation. **(a)** Fractal Ablations. Showing performance between fractal variants: constant, expansion, contraction, and dissociated. **(b)** Fractal SERL returns as a function of grid size. **(c)** Comparison of Fractal SERL vs. Kaleidoscope Experience Replay reflectional symmetries (Lin et al., 2020) for the SIM Reach task. Zoomed in for a better view.

The reach task was conducted in simulation to verify branched symmetries and fractal variants. MuJoCo (Todorov et al., 2012) was used as the physics engine. The state space is composed of six components: $s\left[x_{\mathrm{eff}},\theta ,v,w,g,x_{\mathrm{targ}}\right]$ , the end-effector’s position and orientation, linear and angular velocity, as well as the gripper position and the target block position. The robot reset is fixed, while the block position is randomly sampled. Episodes end after 100 steps. When image-based observations are enabled, the agent additionally receives two RGB images from wrist-mounted cameras, and the explicit block position is omitted. The action space is a three-dimensional delta action a[Δx] . The reward function is dense and defined as $r=\text{clip}\left(e^{-20\cdot d},0,1\right)$ , where d denotes the Euclidean distance between the end-effector and the block. As part of the invariant grid transformations, we use pure translations $\sigma_{t_{k}}\left(\cdot \right)$ . As for the state, these translations $\sigma_{t}\left(s\right)$ only affect positional components $\sigma_{t_{k}}\left(s\right)=s\left[x_{\mathrm{eff}}+t_{k},\theta ,v,w,g,x_{\mathrm{targ}}+t_{k}\right]$ , while the other components (including the hand-centered images) remain unchanged, as explained in Section 4.1.1. With respect to the action space, the translation transformation acts trivially on the action $\sigma_{t_{k}}\left(a\right)=a$ . As such, the same control inputs are executed, but from translated states. In summary, $\sigma_{t_{k}}\left(\tau \right)=\left(\sigma_{t_{k}}\left(s\right),a\right)$ . We perform ablations across different fractal branching strategies and grid sizes and compare them against replay buffer capacity and SymWS size. We also compare against Kaleidoscope Experience Replay (KER) (Lin et al., 2020) to show how branching compares against reflectional symmetries of different orders (Figure 3c).

#### 5.1.1 Branching experiments and results

We tested the performance of fractal branching (constant, expansion, contraction, and dissociated) across five seeds at different grid sizes. We presented the results using both pointwise and training-wide (nAUC).

Although all fractal variations outperformed the baseline, constant branching with a 27×27 grid size performed best. Figure 3b shows how the 27×27 grid outperformed other grid size variations and was used in all subsequent testing.

For pointwise analysis, the learned fetch policy converged reliably to a high-return plateau slightly below the empirical maximum reward (84), consistently solved the reach task, as shown in Figure 3a.

Fractal SERL achieved a return of 75 in 8k steps, whereas SERL took 21k steps, resulting in a 2.6× speedup, or 160%. Our method also achieved peak returns of 81, while SERL reached 75, resulting in approximately an 8% improvement. For trajectory-wide analysis over 50k steps, the Fractal SERL nAUC yielded 0.8803 vs. 0.8116 for SERL, representing an 8% increase.

#### 5.1.2 Branched SERL vs. KER SERL

In this section, we compare the performance of Fractal SERL vs. KER (Lin et al., 2020), which are both built on top of SERL. Originally, KER was trained with 16 reflectional symmetries. Here, we compared [8, 16, 32, 64, 256] reflections. Figure 3c shows that constant branching with Fractal SERL reaches a return of 75 at 4.69 min vs. 8.12 min with KER, representing a 73.1% speed increase or a 0.578 fraction of wall-clock training time.

#### 5.1.3 Grid size vs. replay buffer capacity

Since Fractal SERL fills the replay buffer at a rate of $K^{2}$ , it is imperative to identify a replay buffer capacity that optimizes learning. We varied the grid size and the replay buffer capacity to identify the best-performing configuration. Table 1 summarizes the best hyperparameters.

**TABLE 1:** Optimal replay buffer capacity and symmetric workspace width for varying (K×K) grid sizes for FR3 Sim Reach.

K	$K^{2}$	R.B. Capacity	SymWS (m)
2	4	20K	0.2–0.5
8	64	320K	0.3–0.9
27	729	3.6M	0.2–1.0*
64	4,096	20M	0.2–1.0*

Intuitively, the buffer capacity scales with grid size and indicates the lifetime of each transition (how long before it is replaced) for optimal learning. For FR3 Sim Reach, we found that a capacity of 5,000 transitions (or ∼ 50 episodes) optimized learning.

#### 5.1.4 Grid size vs. symmetry workspace width

As with capacity, the width of the SymWS affects the density of the generated branches and, in turn, learning. Table 1 summarizes identified optimal replay buffer capacities and widths.

Although no simple correlation was found between K and SymWS width, larger grids performed better with larger widths. As the branch granularity decreases, the larger grids perform more robustly. This may be due to the inherent generalization ability of NNs where increased granularity has diminishing returns. From these results, we set a SymWS width of 0.4 m for reach and 0.3 m for peg-insert.

### 5.2 Real FR3 peg-insertion

The FR3 peg-insert task was originally codified by Luo et al. (2024b). Here, a robot inserts a specific-shaped peg into its corresponding slot in a board. The state space includes six components: $s\left[I,x_{\mathrm{eff}},\theta ,v,w,g,x_{\mathrm{targ}}\right]$ , two (128 x 128) images from wrist-mounted cameras, the end-effector’s position and orientation, linear and angular velocities, and the gripper position. The action space is a 7-dimensional space that enacts deltas for position (3), orientation (3), and gripper (1) commands a[Δx,Δθ,Δg] . All actions are constrained within a predefined bounding box to ensure safe execution. We use a sparse reward and terminate episodes upon task completion or 100 steps. As with SERL, we use the impedance controller and relative task frame. We bootstrap the policy with 20 demonstrations and sample 50% from each of the prior and online data replay buffers. We used *random resets* at the start of each episode.

Here, as in the previous experiment, we use pure translations $\sigma_{t_{k}}\left(\cdot \right)$ . For the state, the translations $\sigma_{t}\left(s\right)$ continue to only affect positional components $\sigma_{t_{k}}\left(s\right)=s\left[I,x_{\mathrm{eff}}+t_{k},\theta ,v,w,g\right]$ , while the other components remain unchanged. With respect to the action space, the translation transformation acts trivially on the action $\sigma_{t_{k}}\left(a\right)=a$ . As such, the same control inputs are executed, but from translated states. In summary, $\sigma_{t_{k}}\left(\tau \right)=\left(\sigma_{t_{k}}\left(s\right),a\right)$ . The hyperparameter selection follows that of SERL (Luo et al., 2024a) to maintain equivalent settings, except for hyperparameters specific to Fractal SERL, as shown in Table 2. For this experiment, we used a computer with an AMD Ryzen Threadripper 1950x processor with 16 cores/32 threads running at 3400 MHz, with 128 GB RAM, an Nvidia RTX4070 GPU, and 12 GB of VRAM. Note that baseline work was performed with an RTX4090, which has 2.84 times the FP32 TFLOP capacity (82.6 vs. 29.1) and two times the bandwidth capacity (1008 GB/s vs. 504 GB/s), making our results all the more significant.

**TABLE 2:** Hyperparameters for Fractal SERL.

Parameter	Value
Base SERL/DrQ hyperparameters	
Batch size	256
Discount (γ)	0.96
Optimizer	Adam
Learning rate (actor/critic/temperature)	$3\times 10^{-4}$
Critic ensemble size (E)	10
Critic subsample size	2
Target network EMA weight (ρ)	0.005
Critic-to-actor update ratio	8
Critic network width	[256, 256]
Policy network width	[256, 256]
Layer normalization	Enabled
Initial entropy temperature (α)	$10^{-2}$
Target entropy	−dim(A)/2
Backup entropy in critic target	Disabled
Demo/online replay sampling ratio	50/50
Encoder type	ResNet-10 pretrained
Fractal branching hyperparameters	
Branching method	Constant
Starting branch count (K)	27
Workspace width (SymWS)	0.3
Replay buffer capacity	3,600,000

#### 5.2.1 Ablations and results

**FIGURE 4:**
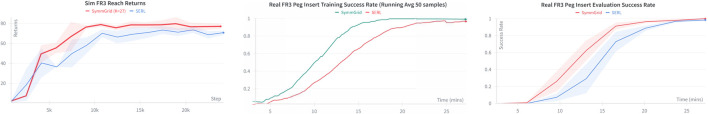
Fractal SERL vs. SERL results obtained directly in a real FR3 peg-insert task. Trained across five seeds and using a constant grid size K=27 . The envelope depicts the standard error of the mean. **(a)** Training success rate vs. wall-clock time. The plot was smoothed by a 50-sample running average. Fractal SERL converges to a97% success rate in 17 min vs. 24.8 min. A 46% speedup in training convergence. **(b)** Evaluation success rate vs. wall-clock time. Fifty insertions attempted for policies trained at different times. Fractal SERL achieves an ∼90% success rate at approximately 15.9 min, while SERL at 20.13 mins, a 27% speedup. **(c)** Real evaluation success rate vs. wall-clock time. Fifty insertions attempted for policies trained at different times. Fractal SERL achieves an ∼90% success rate at approximately 15.9 min, while SERL at 20.13 min, a 27% speedup.

We compared methods using both pointwise and training-wide (nAUC) performance analysis between Fractal SERL and SERL across five seeds. Success rates for training and evaluations (across 50 peg insertion attempts) are shown in Figure 4.

We now present both training and evaluation results. Pointwise analysis revealed that Fractal SERL training converged to a 100% success rate over a 50-sample window at 18 min. The fastest run converged at 14.5 min. This is an outstanding result for direct training on physical robots, as far as we know, in the robotic manipulation community. SERL did not reach a 100% success rate within 27 min of recorded training time. Our version of SERL achieved a top performance of 97% at 24.8 min, while Fractal SERL achieved it at 17 min, representing a 46% (7.8 min) speedup. For evaluation pointwise analysis, Fractal SERL achieved a 90% success rate at 15.9 min, while SERL took 20.13 min, representing a 27% speedup. Similarly, Fractal SERL achieved a 96% success rate at 20 min, while SERL took 23.7 min [the original source cited 60 min with random resets (Luo et al., 2026)], representing a 17% speedup. Fractal SERL also reached a 100% success rate at 27 min. The trajectory-wide nAUC (over 3,500 steps) is particularly important as it allows us to compare the overall performance of the algorithms. Fractal SERL achieved 0.607 compared to 0.499 for SERL, representing a 21.5% increase. Finally, as shown by our narrower standard error envelopes in Figure 4, Fractal SERL yielded more consistent performance.

Fractal evaluations have not yet been conducted directly on the physical robots. Further study is required to evaluate whether they would yield better results and under what conditions. This is left as future work.

### 5.3 Discussion

Fractal SERL learned a robust peg-insert policy in 20 min of wall-clock time with image-based observations, sparse rewards, and random resets by super-scaling symmetric transformations for data generation in a real FR3 robot. Our study provides speedups of up to 46% and 27% vs. baseline for training convergence and evaluation results. We achieve SOTA results in efficient DRL policy learning directly on robots. Fractal SERL also showed superior performance throughout a wide spectrum of training times, with a nAUC 21.5% larger than that of baselines. The potential for further acceleration is promising and will be reported upon integration of a large set of independent compound symmetry transformations, along with potential fractal variations. A disadvantage of Fractal SERL is increased memory usage due to super-scaling. However, with parallelization and specialized image handling in the replay buffer, the increased memory usage becomes negligible. Our study is still preliminary. Further testing is needed across branching strategies for real robots, including integrating possible symmetry combinatorics, testing more challenging tasks, and adding additional baseline comparisons.

## 6 Conclusion

Our Fractal SERL methodology super-scales data generation in real robots by leveraging symmetry branching and training real robot contact-based DRL policies in 20 min on average and as fast as 14 min. Our results significantly advance wall-clock training time efficiency, lowering the barrier to deploying robots and learning directly with them in the wild.

## Data Availability

The datasets presented in this study can be found in online repositories. The names of the repository/repositories and accession number(s) can be found below: https://drive.google.com/drive/folders/1TJlFUiuWsGYZc4kGpnGWGAxe5aRFoEgk?usp&equals;sharing.

## Footnotes

1 A compact set contains all its limit points and is bounded inside a finite region of space
